# Effect of Laser Peening on Microstructural Changes in GTA-Welded 304L Stainless Steel

**DOI:** 10.3390/ma15113947

**Published:** 2022-06-01

**Authors:** Young-Ran Yoo, Jae-Sung Kim, Young-Sik Kim

**Affiliations:** 1 Materials Research Centre for Energy and Clean Technology, Andong National University, 1375 Gyeongdong-ro, Andong 36729, Korea; 2 Chosun Welding Co., Ltd., 34-13, Hwasan 2-gil, Onsan, Ulju, Ulsan 45009, Korea; js2kim@csweld.com; 3 School of Materials Science and Engineering, Andong National University, 1375 Gyeongdong-ro, Andong 36729, Korea

**Keywords:** stainless steel, welding, laser peening, microstructure, hardness

## Abstract

The introduction of tensile residual stress has led to the induction of damage such as fatigue, corrosion fatigue, and stress corrosion cracking (SCC) in stainless steel in association with the influence of environments, components, surface defects, and corrosive factors during its use. Compressive residual stress can be achieved through various techniques. Among several methods, laser peening can be more attractive as it creates regularity on the surface with a high-quality surface finish. However, there is very little research on heavily peened surface and cross-section of stainless steel with very deep compressive residual stress. This work focused on welding and laser peening and the influence of Al coating on the microstructural changes in 304L stainless steel. The specimen obtained by laser peening had a very deep compressive residual stress of over 1 mm and was evaluated based on microstructural and hardness analysis. Therefore, a model for microstructural change by laser peening on welded 304L stainless steel was proposed.

## 1. Introduction

Because stainless steel has good corrosion resistance, its alloys have been used widely in power plants, shipbuilding, marine, railway, aerospace, construction, etc. Technologies such as welding and bonding are widely utilized in these industries. Despite the advancements in technology, welded parts are well-known as the fracture initiation area of the material, which is closely related to the induction of tensile residual stress by the welding process.

The introduction of tensile residual stress has led to the induction of damage such as fatigue, corrosion fatigue, and stress corrosion cracking (SCC) in stainless steel in association with the influence of environment, components, surface defects, and corrosive factors during its use with time [1]. Among the several types of damage, stress corrosion cracking is one of the important failure mechanisms, and many researchers have been working to find a solution to the existing problem.

Stress corrosion cracking is induced by the combination of susceptible materials, tensile stress, and a corrosive environment [2,3,4]. To prevent the formation of SCC, many techniques such as microstructural changes, alloy substitution, elimination of corrosive agents, and induction of compressive stress were utilized. Many studies focused on the induction of compressive residual stress and the following representative methods were employed: shot peening (SP) [5,6,7,8], laser peening (LP) [9,10,11], water-jet peening (WJP) [12,13], ultrasonic peening (UP) [14,15,16], and ultrasonic nanocrystal surface modification (UNSM) [17,18,19]. These technologies plastically deform the surface, refine the microstructure, and reduce tensile residual stress, thereby inducing compressive residual stress, facilitating diffusion via grain boundaries, and enhancing intergranular corrosion resistance and passive properties [20,21,22]. However, the peened surface may act as the initiation site of corrosion due to the formation of mechanically overlapped waves caused by peening [23].

Therefore, laser peening can be more attractive as it can generate surface regularity and a high-quality surface finish [24,25,26]. Laser peening is the cold forming process used to treat the surface, and compressive residual stress can be achieved by the shock wave due to the expansion of high-pressure plasma. In the laser peening process, a focused, high-energy, and short-pulsed laser beam irradiates the surface of a metal specimen, which is usually covered with a thin layer of overlay water [27], glass [28], paint [29], aluminum foil [30,31], or tape [32,33] for protection against melting [28].

The laser-induced compressive residual stress field has various beneficial effects on the microstructure and mechanical and electrochemical properties of the peened metals. Microstructural changes such as dynamic recrystallization and grain refinement help in improving mechanical performance [28,34,35]. The increased dislocation density due to grain refinement has been demonstrated to increase the hardness [36,37,38,39]. In austenitic stainless steel (SS304), air blast shot peening (ABSP), ultrasonic shot peening (UPS), or UNSM peening treatment induces comprehensive deformation microstructures’ grain refinement [40].

There has been very little research on the heavily peened surface and cross-section of stainless steel, which has a very deep compressive residual stress of over 1 mm. This work focused on the effects of laser peening along with factors such as welding and the presence of a thin aluminum layer on the microstructural changes in 304L stainless steel.

## 2. Experimental Methods

### 2.1. Specimen

**Figure 1:**
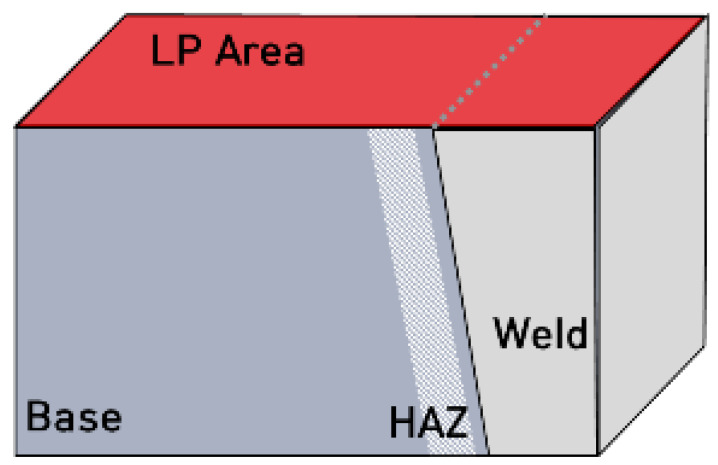
Schematic diagram of the base metal, HAZ, and welded metal specimens.

Commercial 304L stainless steel (304LB) was used in the study and the specimen was welded by the gas tungsten arc welding (GTAW) method (304LW). Table 1 shows the chemical composition of the specimen and its filler metal (ER308L) used in this work. The thickness of the specimen was 25 mm and the groove angle was 15°. Table 2 reveals the welding conditions. In this work, there were many variables; the specimen designation is summarized in Table 3, where HAZ means heat-affected zone. Figure 1 shows a schematic diagram of the base metal, HAZ, and welded metal specimens.

### 2.2. Laser Peening (LP)

**Figure 2:**
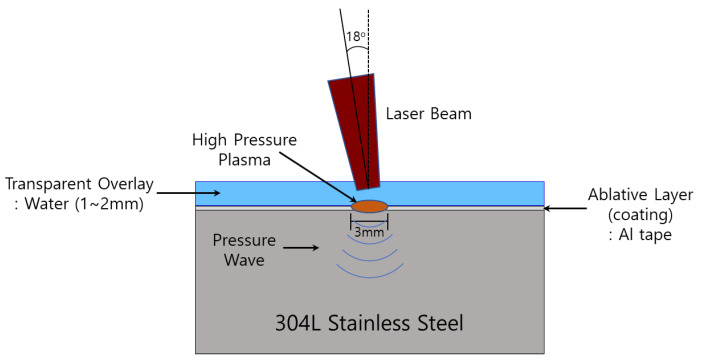
Schematic of laser peening process.

Laser peening was performed using Nd-YAG (1064 nm, IR), and peening conditions were a laser energy of 4.4 J, laser spot diameter of 3 mm, and laser overlay of 50%. Peening was performed utilizing a laser beam with a high-energy pulse generated by an LP generator, which had an impact on the specimen mounted on the jig. Figure 2 shows the schematic diagram on the laser peening process [24]. The detailed parameters of LP are presented in Table 4. The peening parameters for the two types (noncoated/with coating) of LP specimens were the same. However, the specimens differed in the presence of coating with the tape (50 μm Al foil). We mainly used the conditions of 1~2 Hz as the repetition rate, the scanning strategy was about 50% of the overlap ratio, and 1~2 layers (the ablation threshold value of 304L stainless steel is about 0.5~0.7 J/cm^2^ based on 1 pulse).

### 2.3. Optical Microscopic Observation

The specimen was cut to 15 × 15 × 10 mm, ground with #2000 SiC paper, and polished with a 3 µm diamond paste. Etching was performed using an electrolytic etcher (Lectropol-5, Struers, Denmark). As the etching solution, 10% oxalic acid solution was used. Finally, after washing the specimen with ethyl alcohol using an ultrasonic cleaner, the changes in the microstructure were observed with an optical microscope (AXIOTECH 100 HD, ZEISS, Oberkochen, Germany).

### 2.4. Three-Dimensional Microscopic Observation

The surface profile was measured at ×400 magnification using a 3D microscope (KH-7700, HiROX, Tokyo, Japan).

### 2.5. SEM-EDS Analysis

The surface morphology and compositional analysis were performed using a field emission-SEM (MIRA3XMH, Tescan, Brno, Czech Republic) and an energy-dispersive X-ray spectrometer (EDS, Tescan, Brno, Czech Republic), respectively. In the case of the LP-treated specimen, the analysis was performed on the peened surface without surface grinding, but in the case of the nontreated specimen, the surface was ground using a SiC paper until #2000 and mirror-finished before the analysis. In the EDS measurements, electron energies K of 15 KV were provided, working distance was 15 mm, and it was analyzed by fixing the device value for metallic materials.

### 2.6. EBSD Measurement

Specimens for EBSD analysis were polished using #2000 SiC paper and mirror-finished with 1 μm diamond paste. Ion milling was performed for 30 min using an ion-milling machine (IM 4000, Hitachi, Tokyo, Japan).The equipment used for EBSD measurement was electron backscattered diffraction (EBSD, Oxford Instruments, Bognor Regis, UK) attached to FE-SEM (MIRA3 XMH, Tescan, Brno, Czech Republic), and the EBSD step size was 0.3 μm. Data for analysis were postprocessed using HKL Channel 5 (Oxford Ins., Abingdon, UK) analysis software.

### 2.7. Hardness Measurement

After cutting the specimen by a low-speed diamond wheel cutter to 15 × 15 × 10 mm, the specimen was polished using SiC paper to #2000 and then mirror-finished with diamond paste to 1 μm particle size on the cross-section. The hardness was measured in units at 200 μm in depth from the surface using a micro-Vickers hardness tester (HV-100, Mitutoyo, Sakado, Japan).

## 3. Results and Discussion

### 3.1. Microstructural Changes in Surface Due to Welding and Laser Peening

**Figure 3:**
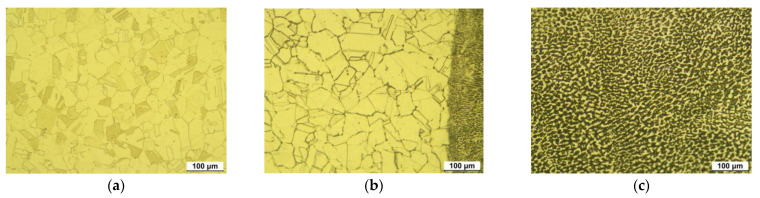
Optical microstructure of 304L stainless steel before LP (OM, ×200, 10% oxalic acid); (**a**) 304LB (base metal), (**b**) 304LW-H (HAZ), and (**c**) 304LW-W (weldment).

Figure 3 shows the optical microstructure on the surface of 304L stainless steel before laser peening. Figure 3a depicts the austenitic microstructure of the base metal and its grain number was calculated as 8.243. Figure 3b reveals the microstructure of the HAZ area and weldment of the specimen. The grain size number of the HAZ area was calculated as 7.826 and it was observed to grow with welding. Figure 3c represents the dual-phase of the weldment due to the precipitation of delta ferrite by GTAW.

**Figure 4:**
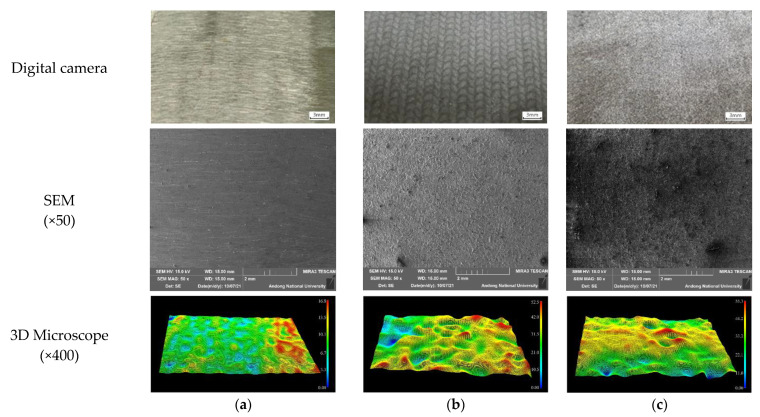
The surface appearance of 304L stainless steel after LP; (**a**) 304L(nonpeened), (**b**) 304L-L-NC (laser-peened without Al coating), and (**c**) 304L-L-WC (laser-peened with Al coating).

Figure 4 shows the surface appearance (digital camera photo, SEM, and 3D microscopy) of 304L stainless steel after laser peening. Figure 4a depicts the surface appearance of a nonpeened specimen. The scratched surface can be observed on the digital camera photo as the surface was ground before laser peeing; however, the low-magnification SEM image and 3D microstructure show a relatively smooth surface compared with the others. The maximum surface roughness measured by a 3D microscopy was 15 μm for nonpeened base metal, 20 μm for nonpeened HAZ area, and 33 μm for nonpeened weldment. Figure 4b presents the surface appearance of the laser-peened specimen without Al coating, and the peened surface can be observed on the digital camera’s photo; however, the low-magnification SEM image and 3D microstructure show a relatively rougher surface than the nonpeened specimen. The maximum surface roughness measured by 3D microscopy was 49 μm for laser-peened base metal, 51 μm for laser-peened HAZ area, and 49 μm for laser-peened weldment. Figure 4c presents the surface appearance of the laser-peened specimen with Al coating, and the peened surface can be observed on the digital camera’s photo; however, the low-magnification SEM image and 3D microstructure show it had a relatively rougher surface than the nonpeened specimen. The maximum surface roughness measured by 3D microscopy was 48 μm for laser-peened base metal, 47 μm for laser-peened HAZ area, and 69 μm for laser-peened weldment. In summary, the laser peening increased the surface roughness, and the peening with Al-coating did not significantly affect the roughness.

**Figure 5:**
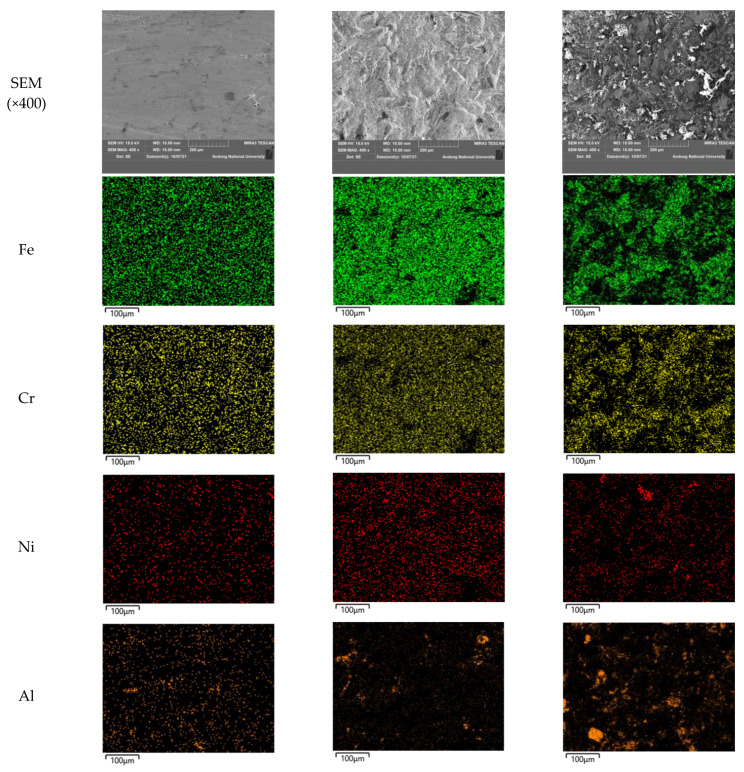

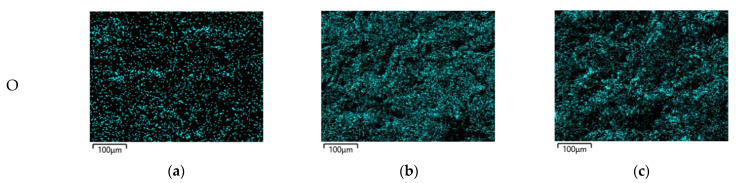
SEM images and elemental distribution on the surface of 304L stainless steel after LP: (**a**) 304L, (**b**) 304L-L-NC, and (**c**) 304L-L-WC.

Figure 5 shows a high-magnification SEM image and elemental distribution on the surface of 304L stainless steel after laser peening. As can be seen in the SEM images, the surface was roughened by laser peening, and the presence of aluminum can be seen in the image. The presence of aluminum can also be confirmed through elemental distribution; however, a small amount of aluminum was observed even in nonpeened and laser-peened surfaces without Al coating due to the contamination of the specimen. In the case of the laser-peened specimen, the increase in surface roughness induced compositional irregularity along with Al concentration on the roughened area, as shown in Figure 5b,c.

**Figure 6:**
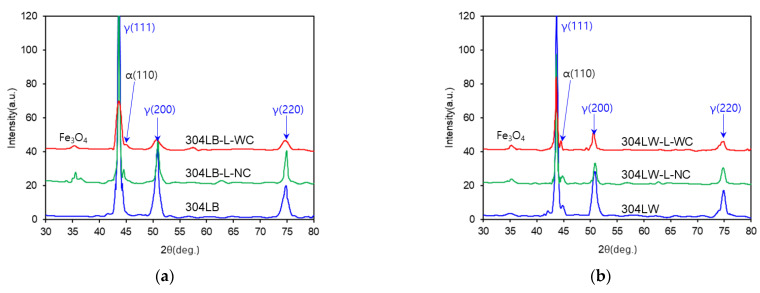
The X-ray diffraction patterns of 304L stainless steel after LP: (**a**) base metal, (**b**) weldment.

We obtained the XRD pattern of the 304L stainless steels for microstructural characterization (Figure 6). From the result of XRD analysis, it mainly contained austenite (γ), the ferrite (α) phase was also slightly detected with the welding, and the Fe_3_O_4_ phase was observed as the laser-peening process was performed with the water film or the welding. However, microstructural variations with the laser-peening process were not observed in this work.

### 3.2. Microstructural Changes in Cross-Section of 304L Stainless Steel by Welding and Laser Peening

**Figure 7:**
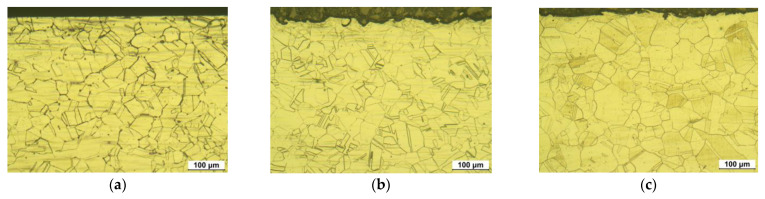
Optical microstructure of the cross-section of 304L base metal after LP (OM, ×200, 10% oxalic acid): (**a**) 304LB, (**b**) 304LB-L-NC, and (**c**) 304LB-L-WC.

Figure 7 shows the optical microstructure of the cross-section of 304L stainless steel. Figure 7a depicts the microstructure of the cross-section of nonpeened base metal. The outermost surface was flat and the grain number was 7.794, thereby signifying the grain size of the cross-section was bigger than the surface. Figure 7b reveals the microstructure of the cross-section of the laser-peened base metal without Al coating. The outermost surface was severely rough and the grain size number was 8.822. Figure 7c represents the microstructure of the cross-section of the laser-peened base metal with Al coating. The outermost surface was severely rough and the grain size number was calculated as 9.115. In summary, laser peening roughened the surface irrespective of the presence or absence the Al coating. In addition, the grain of the cross-section was refined by laser peening, and the Al coating in laser peening was observed to be more effective in causing grain refinement of the base metal.

**Figure 8:**
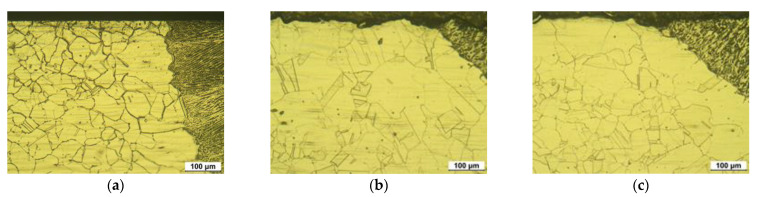
Optical microstructure of the cross-section of 304L HAZ area after LP (OM, ×200, 10% oxalic acid): (**a**) 304LB, (**b**) 304LB-L-NC, and (**c**) 304LB-L-WC.

Figure 8 shows the optical microstructure of the cross-section of GTA-welded 304L stainless steel. Figure 8a depicts the microstructure of the cross-section of the nonpeened HAZ area of a welded specimen.

The outermost surface was flat and the grain size number was calculated as 7.527, thereby signifying that the grain size of the cross-section was bigger than the surface. Figure 8b reveals the microstructure of the cross-section of the laser-peened HAZ area without Al coating. The outermost surface was severely rough and the grain size number was calculated as 8.411. Figure 8c represents the microstructure of the cross-section of the laser-peened HAZ area with Al coating. The outermost surface was severely rough and the grain size number was calculated as 8.589. In summary, the laser peening roughened the welded surface irrespective of the presence or absence of Al coating. The grain of the cross-section was refined by laser peening, and the Al coating in laser peening was observed to be more effective in causing grain refinement of the base metal. However, the welding process led to grain growth irrespective of laser peeing.

**Figure 9:**
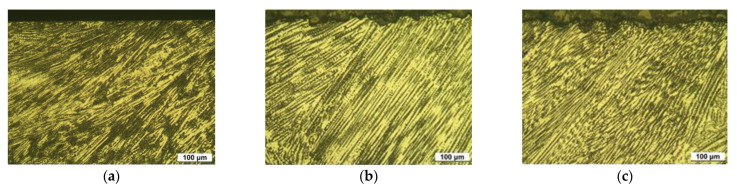
Optical microstructure of the cross-section of 304L weldment area after laser peening (OM, ×200, 10% oxalic acid): (**a**) 304LB, (**b**) 304LB-L-NC, and (**c**) 304LB-L-WC.

Figure 9 shows the optical microstructure of the cross-section of the 304L weldment area with laser peening. The surface was roughened by laser peening, and dendrites of delta ferrite formed along with the heat flow with welding.

**Figure 10:**
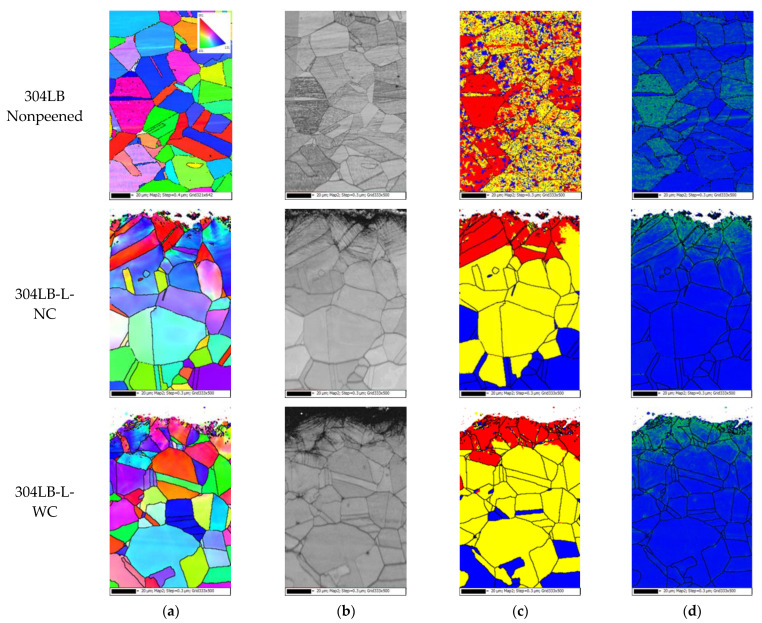
EBSD results of 304L base metal after laser peening (EBSD: step size 0.3 μm, depth ~150 μm): (**a**) IPF coloring, (**b**) band contrast, (**c**) recrystallized fraction, and (**d**) dislocation density.

Figure 10 shows the EBSD results of the 304L base metal with laser peening; the step size was 0.3 μm and the measuring depth was about 150 μm. The inverse pole figure (IPF) coloring in Figure 10a reveals crystal orientation, the band contrast in Figure 10b reveals the grain refinement, the recrystallization fraction in Figure 10c reveals the relative mechanical deformed fraction, and Figure 10d shows the Kernal average misorientation (KAM) and the dislocation density. As can be seen in Figure 10a,b, the outermost surface is extremely grain-refined. As can be seen in Figure 10c, the mechanically deformed area on the outermost surface and the increase in the dislocation density on the outermost area were caused by laser peening.

**Figure 11:**
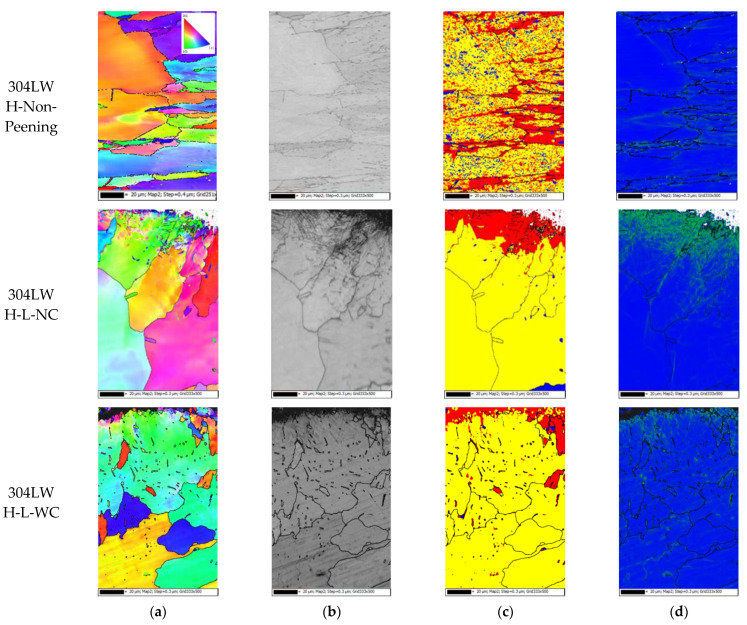
EBSD results of 304L HAZ area after laser peening (EBSD: step size 0.3 μm, depth ~150 μm): (**a**) IPF coloring, (**b**) band contrast, (**c**) recrystallized fraction, and (**d**) dislocation density.

Figure 11 shows the EBSD results of the HAZ area of 304L welded by laser peening; the step size was 0.3 μm and the measuring depth was about 150 μm. In Figure 11a,b, the outermost surface can be seen as extremely grain-refined. Figure 11c reveals the presence of a mechanically deformed area on the outermost surface along with an increase in the dislocation density on the outermost area due to laser peening.

**Figure 12:**
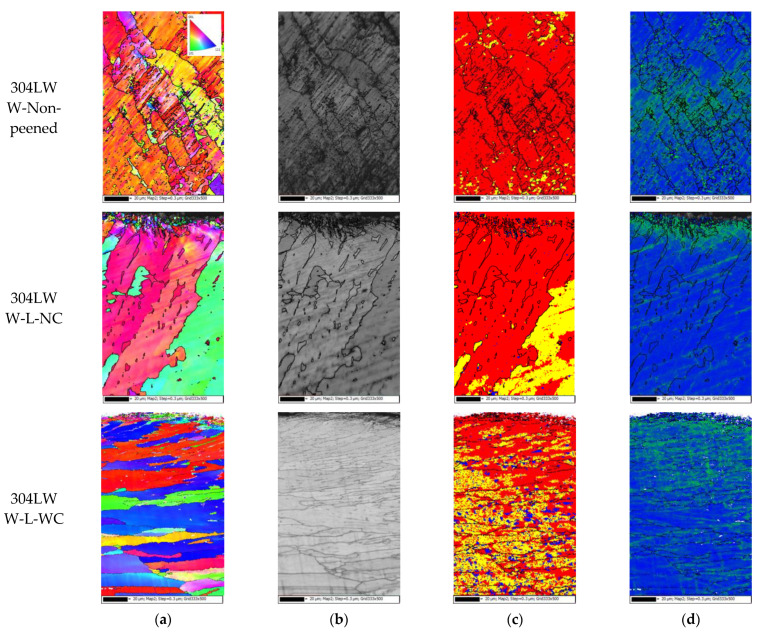
EBSD results of 304L weldment after laser peening (EBSD: step size 0.3 μm, depth ~150 μm); (**a**) IPF coloring, (**b**) band contrast, (**c**) recrystallized fraction, and (**d**) dislocation density.

Figure 12 shows the EBSD results of the weldment area in 304L welded by laser peening; the step size was 0.3 μm and the measuring depth was about 150 μm. Figure 12a,b shows the outermost surface as extremely grain-refined. Due to laser peening, a mechanically deformed area formed toward the interior compared with other specimens, as shown in Figure 12c. The mechanically deformed area in the nonpeened 304L weldment was measured despite nonpeening, and this may be related to the thermal tensile residual stress caused by welding. Dislocation density increased toward the interior due to laser peening. This behavior may have been related to the dendritic microstructure, as shown in Figure 9. This conduct can also be confirmed in terms of hardness in the cross-section of 304L stainless steel.

**Figure 13:**
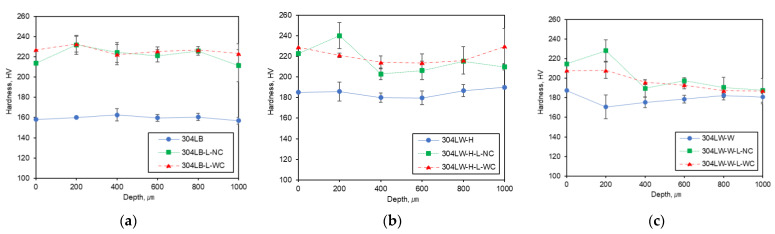
The hardness of the cross-section of 304L stainless steel after LP: (**a**) base metal, (**b**) HAZ, and (**c**) weldment.

Figure 13 shows the Vickers hardness on the cross-section of 304L stainless steel after laser peening. Figure 13a depicts the hardness of the base metal after the peening. The hardness of the nonpeened specimen was low with almost the same value deeper inside. However, the laser peening increased the hardness through the depth and a similar trend can be seen in Figure 10. Figure 13b reveals the hardness of the HAZ area of the welded 304L after the peening. The hardness of the nonpeened specimen was increased by welding with almost the same value deeper inside. However, laser peening extensively increased the hardness of the outermost area with saturation in the depth; a similar trend can be seen in Figure 11. Figure 13c represents the hardness of the weldment area of the welded 304L after the peening. The hardness of the nonpeened specimen was increased by welding with almost the same value deeper inside. However, laser peening extensively increased the hardness of the outermost area with saturation deeper inside. A similar trend can be seen in Figure 12.

**Figure 14:**
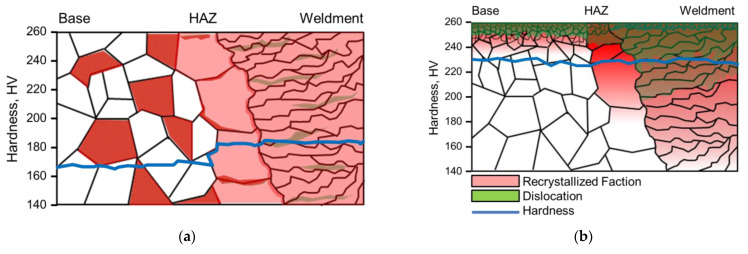
Proposed model of the microstructural variation in 304L stainless steel by (**a**) welding and (**b**) laser peening.

Based on the above observations, a model for microstructural change by laser peening on welded 304L stainless steel was proposed. Figure 14 shows the proposed model for the microstructural variation in 304L stainless steel with welding and laser peening. As shown in Figure 14a, grains grew in the HAZ area and a dendritic microstructure formed by welding, and hardness (blue line) demonstrated a slight increase in the welded area. However, as shown in Figure 14b, laser peening roughened the surface, refined the grain size in the outermost area, caused deformation in the interior of 304L stainless steel, and increased the dislocation density (green) and hardness irrespective of the presence or absence of Al coating. The dendritic microstructure caused by welding facilitated an increase in the dislocation density in the interior of 304L stainless steel during laser peening.

## 4. Conclusions

This study focused on the effects of welding and laser peening and the influence of Al coating on the microstructural changes in 304L stainless steel. The specimen obtained by laser peening had a very deep compressive residual stress of over 1 mm and was evaluated based on microstructural and hardness analysis. The following observations were made:

(1) The grains grew in the HAZ area and a dendritic microstructure formed by welding, and the hardness demonstrated a slight increase in the welded area, irrespective of laser peening.
(2) The laser peening, irrespective of the presence or absence of Al coating, roughened the surface, refined the grain size of the outermost area, deformed the interior, increased the dislocation density, and increased the hardness of 304L stainless steel. The dendritic microstructure formed by welding facilitated the increase in dislocation density in the interior during laser peening.

## Figures and Tables

**Table 1:** Chemical composition of 304L stainless steel and filler metal (wt %).

	C	Cr	Ni	Mn	Si	Cu	Mo	Co	P	N	S	Cb + Ta	Fe
304L	0.02	18.6	9.6	1.65	0.47	-	-	0.03	0.022	0.07	0.03	-	Bal.
ER308L	0.015	19.81	9.84	1.691	0.351	0.115	0.046	0.030	0.024	0.041	0.03	0.008	Bal.

**Table 2:** Welding conditions of the experimental specimen.

Welding Process	Current (A)	Voltage (V)	Speed (cm/min)	Shield Gas (%)	Groove Angle (°)	Welding Electrode
GTAW	245~250	14~15	9~10	Ar. 99.9	15	ER308L (Dia. 0.9 mm wire)

**Table 3:** Designation of the experimental specimen.

Alloy		Nonpeened	Laser Peening	
			Noncoated	With Coating
304L	Base metal	304LB	304LB-L-NC	304LB-L-WC
	HAZ area	304LW-H	304LW-H-L-NC	304LW-H-L-WC
	Weldment	304LW-W	304LW-W-L-NC	304LW-W-L-WC

**Table 4:** Conditions of laser peening treatment.

Laser Type	Laser Energy (J)	Laser Spot Diameter (mm)	Laser Overlay (%)	Transparent Overlay	Laser Incident Beam Angle (°)	Coating
Nd-YAG (1064 nm, IR)	4.4	3	50	Water (1~2 mm)	18	Al tape

## Data Availability

Not applicable.
